# Coenzyme Q10 Supplementation Effects on In Vitro Oocyte Maturation, Lipid Peroxidation, and Embryonic Development in Prepubertal and Aging Thai–Holstein Cows

**DOI:** 10.3390/ani15010018

**Published:** 2024-12-25

**Authors:** Ruthaiporn Ratchamak, Supakorn Authaida, Thirawat Koedkanmark, Wuttigrai Boonkum, Vibuntita Chankitisakul

**Affiliations:** 1 Major of Animal Science, Department of Agricultural Technology, Faculty of Technology, Mahasarakham University, Maha Sarakham 44150, Thailand; ruthaiporn.r@msu.ac.th; 2 Department of Animal Science, Faculty of Agricultural, Khon Kaen University, Khon Kaen 40002, Thailand; supakorn.u@kkumail.com (S.A.); thirawat.koe@kkumail.com (T.K.); wuttbo@kku.ac.th (W.B.); 3 The Research and Development Network Center of Animal Breeding and Omics, Khon Kaen University, Khon Kaen 40002, Thailand

**Keywords:** aging, CoQ10, prepubertal, ovum pick up

## Abstract

This study investigated the effects of coenzyme Q10 (CoQ10) supplementation on in vitro oocyte maturation, lipid peroxidation, and embryonic development in prepubertal and aging Thai–Holstein cows. First, we used slaughterhouse-derived oocytes to confirm that CoQ10 (50 μM) significantly enhanced cleavage (53.33% vs. 37.50%) and blastocyst formation rates (46.81% vs. 27.50%). Thereafter, oocytes were collected from four prepubertal and four aging cows via ovum pick-up and matured in vitro with or without CoQ10 supplementation. The follicular development and oocyte quality were assessed. Aging cows exhibited significantly more follicles (24.00 vs. 16.67) and greater oocyte recovery (16.67 vs. 11.67) than prepubertal cows. Additionally, CoQ10 supplementation significantly reduced malondialdehyde levels in aging cows (1.28 vs. 0.61 nmol/mL), indicating reduced lipid peroxidation. Finally, CoQ10 significantly improved cleavage rates in both age groups (prepubertal: 22.50 to 32.50%; aging: 41.71 to 65.00%) and blastocyst formation rates in aging cows (prepubertal: 17.50 to 20.00%; aging: 31.44 to 53.72%). These results suggest that CoQ10 supplementation enhances oocyte maturation and embryonic development, particularly in aging cows, likely by mitigating oxidative stress and supporting mitochondrial function. Therefore, CoQ10 may be a valuable supplement in assisted reproductive technologies for improving reproductive efficiency in cattle breeding programs.

## Simple Summary

This study explores the impact of Coenzyme Q10 supplementation on in vitro maturation (IVM) of oocytes, lipid peroxidation, and embryonic development in prepubertal and aging Thai–Holstein cows. The findings show that CoQ10 supplementation significantly improves cleavage and blastocyst formation rates, especially in aging cows, by reducing lipid peroxidation levels and mitigating oxidative stress. Prepubertal cows exhibited lower follicular activity and oocyte recovery rates compared to aging cows, which had a higher number of larger follicles and superior-quality oocytes. These results are particularly valuable for assisted reproductive technologies (ARTs) aimed at increasing the efficiency of cattle breeding programs. By improving oocyte competence, especially in aging cows with valuable genetics, this research contributes to extending the productive lifespan of these animals and addressing fertility challenges associated with aging. Consequently, CoQ10 supplementation offers a promising avenue for enhancing ART success and bolstering genetic advancements in the cattle industry.

## 1. Introduction

Age-related declines in bovine fertility are a significant challenge for efficient cattle production. This decrease is partly attributed to increased oxidative stress, which impairs oocyte quality and embryonic development [1]. Assisted reproductive technologies (ARTs) such as ovum pick-up (OPU) offer strategies to improve reproductive efficiency; however, the success of these techniques is often compromised by the reduced developmental competence of oocytes from older cows [2]. The use of OPU to collect oocytes from cows of various ages presents an important opportunity to advance reproductive technologies and improve genetic traits in bovine species [3]. Studying oocytes from prepubertal cows can accelerate genetic progress via the early use of high-value genetics, thereby enhancing breeding programs [3]. Conversely, oocytes from aging cows (>8 years) may be used to address the challenge of declining fertility associated with advanced age [4]. Despite their reduced reproductive efficiency, older cows often possess valuable genetic traits that can be preserved through embryo production, maximizing their genetic contributions even in their later years. Therefore, evaluating the quality and quantity of oocytes from different age groups is important for improving breeding practices and increasing animal productivity.

Coenzyme Q10 (CoQ10) is a potent antioxidant that plays a crucial role in mitochondrial function and is essential for oocyte maturation and embryonic development. CoQ10 supplementation mitigates oxidative stress and improves reproductive outcomes in various animal models [5,6,7]. Its antioxidant properties make it a promising candidate for oocyte and embryo quality improvement in aging cows, which experience higher levels of oxidative stress due to suboptimal in vitro maturation (IVM) conditions. Increased oxidative stress leads to reduced oocyte quality, lower cleavage rates, and decreased blastocyst formation due to cellular stress, metabolic imbalances, and oxidative damage [8,9,10]. Excessive generation of reactive oxygen species (ROS), a byproduct of cellular metabolism in the IVM environment, can result in oxidative damage to lipids, proteins, and DNA within oocytes, ultimately impairing mitochondrial function and embryonic development [11,12,13,14]. The female reproductive system possesses a range of natural antioxidants to protect oocytes and embryos from ROS; nevertheless, in vitro-produced embryos reportedly have substantially lower antioxidant levels than those observed in vivo [15].

Therefore, this study investigated the differential responses of ovarian follicular growth on follicular aspiration (OPU) in prepubertal and aging cows. Additionally, we evaluated the hypothesis that CoQ10 supplementation during IVM enhances oocyte developmental competence in both prepubertal and aging Thai–Holstein cows. We assessed the effects of CoQ10 on lipid peroxidation (a marker of oxidative stress) as well as the cleavage and blastocyst formation rates to determine whether CoQ10 could mitigate the negative effects of aging on oocyte quality and embryonic development. This study may contribute to the improvement of ART success rates and enhancement of reproductive efficiency in the cattle industry.

## 2. Materials and Methods

### 2.1. Experimental Design

This study investigated the effects of CoQ10 supplementation on oocyte developmental competence using two approaches: in vitro embryo development of slaughterhouse-derived oocytes (Experiment 1) and in vivo evaluation of oocyte quality and developmental potential in prepubertal and aging Thai–Holstein cows (Experiment 2).

*Experiment 1: Impact of CoQ10 Supplementation of IVM Medium on Development of Embryos from Slaughterhouse-Derived Oocytes*

This experiment assessed the effectiveness of CoQ10 supplementation in improving the cleavage and blastocyst formation rates of oocytes derived from slaughterhouse ovaries. In alignment with our preliminary findings and those reported by [4], which demonstrated the benefits of CoQ10 in enhancing the oocyte survival rate after vitrification, we assigned a CoQ10 concentration of 50 μM in this study. Cumulus–oocyte complexes (COCs) retrieved from a slaughterhouse were subjected to maturation in IVM medium supplemented (*n* = 70, CoQ10 group) or not supplemented with 50 μM CoQ10 (*n* = 72, control group) for 22 h. Following maturation, those were fertilized and cultured for 8 days. Cleavage and blastocyst formation rates were assessed on Day 2 and Day 7 after insemination, respectively. This experiment was replicated five times.

*Experiment 2: Effect of CoQ10 Supplementation on Oocyte Quality and Developmental Competence in Prepubertal and Aging Thai–Holstein Cows using an Ovum Pick-up (OPU) Procedure*

To establish baseline differences in ovarian function between age groups, follicular development and oocyte recovery were compared in four prepubertal and four aging Thai–Holstein cows. Estrus synchronization was achieved using the hormonal protocol described by Garcia et al. [16]. On day 5 of the synchronization protocol, ovarian follicles were visualized using a real-time ultrasound scanner (Hs-2100V, Honda Electronics, Tokyo, Japan) equipped with a 17-gauge × 500 mm cow ova vacuuming needle (Kitazato, Tokyo, Japan) prior to each OPU. The follicles in the ovaries were counted using ultrasound video images. All visible follicles were quantified and classified according to their diameters: small follicles (<4 mm), medium follicles (4 to 6 mm), and large follicles (>6 mm). Then the follicles were aspirated. Recovered COCs were evaluated and morphologically classified into one of the three following categories: (1) Grade A, >4 layers of cumulus cells; (2) Grade B, 3–4 layers of cumulus cells; or (3) Grade C, 1–2 layers of cumulus cells and denuded oocytes. The OPU sessions were conducted a total of ten times per donor, with each session performed at 14-day intervals to minimize the effects of repeated OPU.

All COCs from each group except Grade C were used for maturation in accordance with Chaubal et al. [17]. The COCs were randomly assigned to IVM medium supplemented with 50 μM CoQ10 (*n* = 163; prepubertal = 80, aging = 83) or not supplemented (control group, *n* = 152; prepubertal = 80, aging = 72) for 22 h. Thereafter, lipid peroxidation was assessed using the thiobarbituric acid reactive substances (TBARS) assay. Key developmental parameters, including cleavage and blastocyst formation rates, were determined and compared between the treatment and control groups.

### 2.2. Slaughterhouse Oocyte Recovery

Ovaries were retrieved from a local slaughterhouse between 09:00 and 10:00 h to obtain oocytes for IVM and fertilization in Experiment 1. The ovaries were transported to the laboratory within an hour of retrieval in modified Dulbecco’s Phosphate Buffered Saline (mDPBS) at 37 °C. Subsequently, the ovaries were thoroughly washed in mDPBS at least three times to remove debris and immediately processed. The time from slaughterhouse collection to initiation of IVM did not exceed two hours. Oocytes were aspirated from antral follicles ranging from 2 to 8 mm in size using an 18-gauge needle connected to a 10 mL syringe. Only COCs with homogeneous cytoplasm and those surrounded by multiple compact layers of cumulus cells were selected for IVM. These COCs, exhibiting characteristics associated with high developmental potential, were identified and collected under a stereomicroscope (Olympus SZ40; Olympus, Tokyo, Japan) and stored in a collection medium (mDPBS containing 0.1% polyvinyl alcohol) until the entire collection procedure was complete.

### 2.3. Animals, Housing Conditions and Ethical Consideration

All the animals were managed in accordance with ethical guidelines for animal welfare. This study was approved by the Institutional Animal Care and Use Committee based on the Ethics of Animal Experimentation of the National Research Council of Thailand (IACUC-KKU-124/66; reference no. 660201.2.11/638(144)).

Eight Thai–Holstein crossbreeds were used in Experiment 2, comprising four prepubertal (<12 months) and four aging cows (8–10 years). All cows had body condition scores ranging from 3 to 3.5 on a 1–5 scale, with average scores of 2.5–3.0 for prepubertal and 3.5 for aging cows. The animals were maintained in straw and fed twice daily in accordance with the requirements for Holstein cows outlined by the National Research Council [18], including a balanced diet of forage and concentrate. All the cows had free access to water and mineral salt.

### 2.4. Chemicals and Media

All chemicals used in the present study were obtained from Sigma-Aldrich (St. Louis, MO, USA) unless otherwise stated. The CoQ10 solution was prepared by dissolving CoQ10 in 2.5% ethyl alcohol, as previously described [19]. The mDPBS medium comprised the following: 100 mL DPBS (D1408-500ML), 1.0 g glucose (G7528-250G), 0.036 g sodium pyruvate (P2256-25G), 0.1 g calcium chloride dihydrate (793639-500G), 0.1 g magnesium chloride hexahydrate (63068-250G), and 1.0 mL penicillin-G (A5955-100ML), all dissolved in 1000 mL of deionized water. Culture media used in this study (BO-Wash, BO-IVM, BO-Semen prep, BO-IVF, and BO-IVC) were obtained from IVF Bioscience (Falmouth, Cornwall, UK).

### 2.5. Estrus Synchronization of Donor Cows and Collection of Immature Oocytes by OPU

To obtain immature oocytes from donor cows for IVM, estrus synchronization was induced by hormone treatment according to the methods described by Garcia et al. [16]. On a random day of the estrous cycle (designated day 0), cows were fitted with an intravaginal progesterone-releasing device (CIDR^®^; Zoetis Animal Health, Kalamazoo, MI, USA) and administered with an intramuscular injection of 2 mg estradiol benzoate (March Pharmaceutical, Bangkok, Thailand) and 25 mg prostaglandin F2α (Lutalyze, Zoetis Animal Health, Kalamazoo, MI, USA). On the morning of day 5, the CIDR device was removed to allow for the development of follicles to a stage suitable for OPU.

Follicular aspiration was performed on day 5 of the synchronization protocol using ultrasonic guidance procedures, with a real-time ultrasound system (Hs-2100V, Honda Electronics, Tokyo, Japan) to guide needle placement and ensure accurate follicular targeting. The OPU protocol was conducted ten times at 14-day intervals.

### 2.6. Ovum Pick-Up

Transvaginal OPU was performed to obtain immature oocytes for IVM and fertilization. The cow was placed in a standing position and restrained using a cattle crush to ensure safety and minimize stress. Transvaginal OPU was conducted as previously described by Chasombat et al. [20], using a 7.5 MHz micro-convex probe (HCV-4710MV; Honda Electronics, Aichi, Japan) coupled with a 17-gauge × 500 mm cow ova vacuuming needle (Kitazato, Tokyo, Japan) connected to a 1500 mm polyvinyl chloride tube linked to an ultrasonic scanner (Hs-2100V, Honda Electronics, Tokyo, Japan). Follicles >3 mm in diameter were aspirated using a real-time ultrasound system to guide needle placement and ensure accurate follicular targeting. A relatively high vacuum pressure of 75 mmHg was used for aspiration to optimize oocyte recovery while minimizing follicular tissue damage. The aspirated follicular contents were collected in a 50 mL conical tube containing approximately 5 mL of mDPBS supplemented with 2% (*v*/*v*) fetal bovine serum (1906286; Biological Industries, Beit HaEmek, Israel) and 100 μL per 10 mL of heparin (heparin injection 5000 IU/mL; GIS Pharma Ltd., Bangkok, Thailand). The collection medium was maintained at 37 °C using a heating block. Subsequently, the sedimented materials in the conical tubes were transferred to 90 mm Petri dishes, and the remaining solution was filtered using a Nipro cell filter (Fujihira Industry Co., Ltd., Tokyo, Japan). COCs with expanded cumuli were identified and collected under a stereomicroscope as they exhibited characteristics associated with high developmental potential. These COCs were stored in a collection (holding) medium (ABT Holding; Pullman, Washington, DC, USA) until the entire collection procedure was complete.

### 2.7. In Vitro Maturation

The selected COCs underwent IVM to induce meiosis completion and prepare them for fertilization. The COCs were further cleaned by successive washes in BO-Wash medium to remove any remaining debris or contaminants. Subsequently, groups of 10 COCs were cultured in 50 μL droplets of BO-IVM medium covered with mineral oil. The COCs underwent IVM for 22 h at 38.5 °C in a humidified atmosphere of 5% CO_2_ in air.

### 2.8. Sperm Preparation, In Vitro Fertilization, and In Vitro Culturing

Frozen semen from dairy bulls was thawed and prepared for in vitro fertilization (IVF) using Percoll gradient centrifugation. The semen was thawed at 37 °C for 30 s and centrifuged in a 3 mL 90% Percoll gradient medium at 328× *g* for 5 min at room temperature. Thereafter, the sperm pellet was washed twice with 2 mL of BO-Semen prep at 328× *g* for 5 min at room temperature. The final sperm concentration was adjusted to 3 × 10⁶ sperm per mL, which is the optimal concentration for IVF in our system.

Following 22 h of IVM, groups of 10 COCs were fertilized with sperm in 50 μL droplets of BO-IVF. In vitro fertilization was conducted for 6 h at 38.5 °C in a humidified atmosphere of 5% CO_2_ in air. Excess cumulus and sperm cells were removed by repeated pipetting in an in vitro culture medium (BO-IVC) to ensure optimal conditions for embryonic development. The presumptive zygotes were washed twice before being transferred to culture in 50 μL droplets of BO-IVC for 48 h. Subsequently, the culture medium was replaced with 50 μL droplets of BO-IVC, and the embryos were cultured for 5–6 more days (7–8 days post-fertilization). All culturing was performed at 38.5 °C in a humidified atmosphere of 5% CO_2_ in air.

Cleavage rates were assessed 48 h after insemination, and the embryos were examined under a stereomicroscope to evaluate development. The percentage of blastocysts was recorded on day 7. Cleavage and blastocyst rates were recorded. Cleavage rate is the percentage of cultured oocytes that underwent successful cell division after fertilization. Blastocyst rate is the percentage of cultured oocytes that developed into blastocysts. Both rates are calculated by dividing the number of successful embryos (either cleaved or blastocysts) by the total number of oocytes initially cultured and then multiplying by 100%.

### 2.9. Measurement of Lipid Peroxidation in IVM Medium

Lipid peroxidation, an indicator of oxidative stress, was measured in the IVM medium using the TBARS reaction to assess oxidative stress levels during IVM [21]. IVM medium samples of 0.25 mL each were incubated with 0.25 mL of 1 mM ascorbic acid and 0.25 mL of 0.2 mM ferrous sulfate to promote lipid peroxidation and generate MDA. The samples were incubated at 37 °C for 60 min in a water bath to facilitate the reaction. Thereafter, 1 mL of trichloroacetic acid (15% [*w*/*v*]) was added to precipitate the proteins and stop the reaction. Subsequently, 1 mL of thiobarbituric acid (0.375% [*w*/*v*]) was added and reacted with MDA, a major product of lipid peroxidation, to form a pink adduct. The mixture was boiled in water at 100 °C for 10 min to enhance color development. After cooling the samples to 4 °C to terminate the reaction, they were centrifuged at 4000× *g* for 10 min at 4 °C and analyzed using a UV-visible spectrophotometer (Specord 250 plus; Analytik Jena, Jena, Germany). The absorbance of the samples was measured at 532 nm, and the MDA concentration (expressed in nmol/mL) was calculated using a standard curve generated with known concentrations.

### 2.10. Statistical Analyses

Analysis of variance (ANOVA) was performed using the GLM procedure in SAS software version 9.0 (SAS Institute, Cary, NC, USA). A priori power analysis was performed using G*Power for each General Linear Model (GLM) to estimate the sample size required to achieve a minimum of 80% statistical power for detecting effect size differences between treatment groups.

In Experiment 1, the effect of CoQ10 supplementation (two treatment groups: CoQ10, *n* = 70; and control, *n* = 72) on the cleavage and blastocyst formation rates of slaughterhouse-derived oocytes was analyzed using a completely randomized design (CRD).

In Experiment 2, the effect of cow age (prepubertal, *n* = 4; aging, *n* = 4) was evaluated as a source of variation in oocyte quality, assessed via follicle number, follicle size, recovery rate, number of oocytes, and oocyte grade. A CRD with ten replicates per cow was employed. The effects of CoQ10 supplementation on lipid peroxidation, cleavage rates, and blastocyst formation rates were analyzed using a CRD with ten replicates per treatment group (control vs. supplemented), controlling for cow age.

In all experiments, Tukey’s post hoc test was used for pairwise comparisons of means, with the level of significance set at *p* ≤ 0.05.

## 3. Results

### 3.1. Experiment 1: Impact of CoQ10 Supplementation of IVM Medium on Development of Embryos from Slaughterhouse-Derived Oocytes

**Figure 1:**
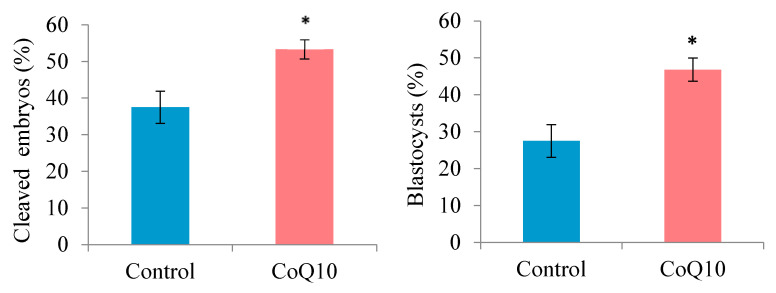
Effects of coenzyme Q10 (CoQ10) treatment on cleavage and blastocyst formation rates of oocytes derived from slaughterhouse ovaries. * *p* < 0.05.

CoQ10 supplementation during IVM significantly improved both cleavage and blastocyst formation rates of oocytes derived from slaughterhouse ovaries. As shown in Figure 1, oocytes treated with CoQ10 yielded a greater percentage of cleaved embryos than those in the control group (53.33 ± 2.64% vs. 37.50 ± 4.41%; *p* = 0.0013), as well as a notable improvement in blastocyst formation (46.81 ± 3.13% vs. 27.50 ± 4.40%, *p* = 0.0006).

### 3.2. Experiment 2: Effect of CoQ10 Supplementation on Oocyte Quality and Developmental Competence in Prepubertal and Aging Thai–Holstein Cows Using an Ovum Pick-Up (OPU) Procedure

This experiment compared the follicular development and oocyte recovery between prepubertal and aging cows. As shown in Table 1, the aging group exhibited significantly more follicles (*p* = 0.0202, 24.00) than the prepubertal group (16.67). Furthermore, follicle size distribution differed between the groups, with smaller follicles (<4 mm) being more prevalent in prepubertal cows (32.10%) than in aging cows (19.53%, *p* = 0.0100). In contrast, larger follicles (>6 mm) were more common in aging cows (47.55 vs. 21.98%, *p* = 0.0276).

Oocyte recovery rates were higher in aging cows, which yielded more oocytes per aspiration (16.67 vs. 11.67, *p* = 0.0402). Moreover, aging cows produced a significantly higher proportion of Grade-A oocytes (44.31%) than prepubertal cows (25.15%, *p* = 0.0395), whereas Grade-B oocytes were more prevalent in the younger group (44.70 vs. 28.99%, *p* = 0.0323).

The effects of CoQ10 supplementation on oxidative stress (as measured by MDA levels) and embryonic development of oocytes obtained via OPU from prepubertal and aging cows were shown in Table 2. CoQ10 significantly reduced MDA levels in both groups, indicating a reduction in lipid peroxidation and oxidative stress. This effect was particularly pronounced in aging cows, in which MDA levels decreased from 1.28 to 0.61 nmol/mL (*p* = 0.0013). A marginal decrease was observed in prepubertal cows (1.04 to 1.03 nmol/mL; *p* = 0.9412).

CoQ10 supplementation also led to significant cleavage rate increases in both groups, with prepubertal cows improving from 22.50 to 32.50% (*p* = 0.0331) and aging cows demonstrating a more substantial increase from 41.71 to 65.00% (*p* = 0.0006). Blastocyst formation followed a similar pattern; CoQ10 significantly enhanced blastocyst formation rates in aging cows (31.44 to 53.72%, *p* = 0.0003), although the increase in prepubertal cows was not significant (17.50 to 20.00%, *p* = 0.4512).

## 4. Discussion

In this study, CoQ10 supplementation during IVM significantly enhanced the embryonic developmental outcomes of bovine oocytes, particularly by affecting the cleavage and blastocyst formation rates. Oocytes from both prepubertal and aging cows demonstrated increased developmental competence following CoQ10 treatment, as evidenced by significant improvements in cleavage rates and blastocyst yields. These effects were likely due to the antioxidant properties of CoQ10, which reduced oxidative stress, as indicated by decreased lipid peroxidation (MDA levels). We presume that the reduced oxidative stress contributed to maintaining cellular integrity and supporting mitochondrial function, particularly in oocytes from aging cows in which oxidative damage is typically more pronounced. This study also highlighted differences in ovarian follicular growth, oocyte recovery and quality, and embryonic development between prepubertal and aging cows. Aging cows had a higher total follicle count as well as an increased recovery rate of oocytes per aspiration than their prepubertal counterparts. Additionally, aging cows produced a higher proportion of Grade-A oocytes, suggesting greater developmental potential. These results underscore the reproductive physiological differences between age groups and suggest that, while prepubertal cows do benefit from CoQ10 supplementation, aging cows experience more pronounced improvements, which potentially reflects age-related mitochondrial deficiencies counteracted by CoQ10.

This study revealed significant differences in follicular dynamics between prepubertal and aging cows. Aging cows had a greater number of follicles and proportion of larger follicles (>6 mm) than their prepubertal counterparts (Table 1). These results are similar to those reported for Japanese Black and German Simmental cows [22,23]. The increase in follicle number and size can be attributed to various physiological changes that occur as cows mature. Aging in cattle is associated with elevated concentrations of gonadotropins such as follicle-stimulating hormones, which may increase the recruitment and development of larger follicles [24]. Larger follicles provide a more favorable environment for oocyte development, affording more space, nutrients, and beneficial factors such as glucose and antioxidant enzymes [25], resulting in elevated cleavage and blastocyst rates [26]. In contrast, prepubertal cows in the present study had fewer follicles and a greater proportion of smaller follicles (<4 mm), reflecting the immaturity of their reproductive systems and underdeveloped ovarian reserves [8].

The significantly reduced MDA levels in the IVM medium of the CoQ10-treated group of aging but not prepubertal cows highlights the heightened susceptibility to oxidative stress associated with aging [27]. Aging oocytes exhibit diminished antioxidant capacity and increased ROS production, largely due to mitochondrial dysfunction, which augment lipid peroxidation and cellular damage [3,28,29]. The observed decrease in MDA levels following CoQ10 supplementation suggests that CoQ10 effectively mitigates age-related oxidative stress by enhancing the antioxidant defenses of the oocytes and reducing ROS-mediated lipid peroxidation [30,31]. In contrast, prepubertal bovine oocytes, which possess relatively higher natural antioxidant levels and lower baseline ROS levels, did not show significant MDA reduction. Our findings suggest a reduced need for exogenous antioxidant protection in prepubertal oocytes compared to those from older animals. This may reflect a difference in intrinsic antioxidant capacity or basal oxidative stress levels. [8].

Reducing oxidative stress during IVM preserves the structural and functional integrity of oocytes, leading to improved cleavage and blastocyst formation rates, particularly in aging cows [9]. The dual role of CoQ10 as an antioxidant and a facilitator of mitochondrial adenosine triphosphate synthesis supports cellular energy demands during IVM, thus creating an optimal environment for embryonic development [5,7]. Previous studies have shown that antioxidants such as CoQ10 improve embryo quality by stabilizing mitochondrial function and protecting cellular components from ROS damage [5]. The current study aligns with these findings, underscoring that CoQ10 supplementation during IVM enhances embryonic competence, particularly in oocytes prone to oxidative stress.

This study revealed significantly lower blastocyst formation efficiency following OPU in prepubertal cows (approximately 20%) than in aging cows (approximately 50%, Table 2). Reduced developmental competence in prepubertal animals is not limited to bovine species [32,33,34]; ovine [35], caprine [36], and swine [37] species also exhibit significantly lower blastocyst yields during prepuberty. This is likely due to the immature physiological and endocrine environments of prepubertal oocytes. Furthermore, COCs from larger follicles typically exhibit greater developmental competence than those from smaller follicles [38]. This is consistent with the findings of Experiment 2, in which older cows had significantly larger follicles and higher-grade oocytes. Similarly, in prepubertal goats, COCs from larger follicles yielded blastocyst formation rates comparable to those of adult animals [39]. These observations are consistent with the established positive correlation between larger follicle size and superior oocyte maturation [25,26]. The smaller size and lower initial metabolic rates of prepubertal oocytes [40] likely contributed to their reduced developmental potential, resulting in lower cleavage and blastocyst formation rates than aging cows identified in this study. Conversely, the larger follicles identified in aging cows were associated with superior oocyte quality and developmental competence, as shown in Table 1 and Table 2. The higher quality associated with larger follicles [41,42] further underscores age-related differences in oocyte competence. Moreover, studies examining gene expression have revealed inferior quality markers and lower expression levels of key genes associated with embryonic development in embryos derived from prepubertal donors [43]. Hence, reduced developmental competence in prepubertal animals may be attributed to insufficient protein translation, incomplete cytoplasmic maturation, and inadequate hormonal stimulation, all of which are crucial for optimal oocyte quality and in vitro development [44]. Therefore, while prepubertal cows offer the potential for genetic advancement, their oocytes exhibit reduced developmental competence compared with those of adult cows.

## 5. Conclusions

This study demonstrates the beneficial effects of CoQ10 supplementation during IVM on oocyte and embryonic development in both prepubertal and aging Thai–Holstein cows. The significant reduction in MDA levels identified in aging cows treated with CoQ10 highlights its antioxidant properties and ability to mitigate age-related oxidative stress. The improvements observed in cleavage and blastocyst formation rates, particularly in aging cows, further confirm the beneficial effects of CoQ10 on oocyte developmental competence. Moreover, we determined that aging cows exhibited significantly greater follicular activity and superior oocyte quality than prepubertal cows. These findings have practical implications for reproductive programs as they suggest that aging cows can still be valuable oocyte donors if appropriately managed. The enhanced follicular activity in aging cows coupled with the use of antioxidants such as CoQ10 during IVM may help preserve oocyte quality and improve embryo production outcomes, thereby extending the productive lifespan of genetically valuable cows.

## Figures and Tables

**Table 1:** Ovarian follicular growth upon ovum pick-up on day 5 of the synchronization protocol in prepubertal and aging Thai–Holstein cows.

Parameter	Prepubertal Cows	Aging Cows	SEM	*p*-Value
Number of follicles	16.67 ^b^	24.00 ^a^	2.19	0.0202
Follicle size distribution (%)				
<4 mm	32.10 ^a^	19.53 ^b^	4.51	0.0100
>4–6 mm	45.91	32.90	5.13	0.1604
>6 mm	21.98 ^b^	47.55 ^a^	5.49	0.0276
Recovery rate (%)	70.47	70.95	4.28	0.9531
Number of oocytes	11.67 ^b^	16.67 ^a^	1.36	0.0402
Grade of oocytes				
Grade A (%)	3.33 (25.15) ^b^	7.67 (44.31) ^a^	4.27	0.0395
Grade B (%)	5.00 (44.70) ^a^	5.00 (28.99) ^b^	3.84	0.0323
Grade C (%)	3.67 (30.15)	4.64 (26.70)	1.34	0.3464

^a,b^ Within each row, means with different superscripts differ significantly (*p* < 0.05) according to Tukey’s post hoc test. SEM, standard error of the mean.

**Table 2:** Malondialdehyde levels in maturation medium and developmental competence of prepubertal and aging bovine oocytes matured with (−) and without (+) coenzyme Q10 supplementation.

Animals	CoQ10 During IVM	Total Oocytes ^1^ (n)	MDA (nmol/mL)	Cleavage ^2^ (%)	Blastocyst Formation ^2^ (%)
Prepubertal cows	−	80	1.04	22.50 ^b^	17.50
	+	80	1.03	32.50 ^a^	20.00
		SEM	0.04	1.95	1.34
		*p*-value	0.9412	0.0331	0.4512
Aging cows	−	72	1.28 ^a^	41.71 ^b^	31.44 ^b^
	+	83	0.61 ^b^	65.00 ^a^	53.72 ^a^
		SEM	0.11	4.44	4.26
		*p*-value	0.0013	0.0006	0.0003

^a,b^ Values with different superscripts within each column and age group differ significantly (*p* < 0.05). ^1^ Grade-A and -B oocytes retrieved for maturation ^2^ Based on oocyte maturation CoQ10, coenzyme Q10; IVM, in vitro maturation; MDA, malondialdehyde; SEM, standard error of the mean.

## Data Availability

The data are available upon request from the corresponding author.
